# Low-Cost Electronics for Automatic Classification and Permittivity Estimation of Glycerin Solutions Using a Dielectric Resonator Sensor and Machine Learning Techniques

**DOI:** 10.3390/s23083940

**Published:** 2023-04-12

**Authors:** Miguel Monteagudo Honrubia, Javier Matanza Domingo, Francisco Javier Herraiz-Martínez, Romano Giannetti

**Affiliations:** Institute for Research in Technology, ICAI School of Engineering, Comillas Pontifical University, 28049 Madrid, Spain; jmatanza@iit.comillas.edu (J.M.D.); fjherraiz@icai.comillas.edu (F.J.H.-M.)

**Keywords:** dielectric resonator, microwave sensor, machine learning, dielectric characterization, glycerin purification, low-cost electronics, arduino

## Abstract

**Simple Summary:**

Glycerin is an organic substance used as an ingredient for many industries, including pharmaceuticals and cosmetics, but also, glycerin is an important product during biodiesel refining. Accurate and real-time sensors are needed to improve the industrial process; therefore, we proposed a workflow to measure concentrations of glycerin using a microwave sensor enhanced by machine learning models. We tested this methodology with complex electronic instrumentation and a designed low-cost portable electronic reader. As a result, we found that both devices achieved excellent and similar performance. These findings are valuable since monitoring the glycerin concentration may help to increase efficiency and reduce costs in the industry. In addition, the methodology proposed in this study could be applied to any sensor, making it a valuable contribution to liquid analysis with microwave sensors.

**Abstract:**

Glycerin is a versatile organic molecule widely used in the pharmaceutical, food, and cosmetic industries, but it also has a central role in biodiesel refining. This research proposes a dielectric resonator (DR) sensor with a small cavity to classify glycerin solutions. A commercial VNA and a novel low-cost portable electronic reader were tested and compared to evaluate the sensor performance. Within a relative permittivity range of 1 to 78.3, measurements of air and nine distinct glycerin concentrations were taken. Both devices achieved excellent accuracy (98–100%) using Principal Component Analysis (PCA) and Support Vector Machine (SVM). In addition, permittivity estimation using Support Vector Regressor (SVR) achieved low RMSE values, around 0.6 for the VNA dataset and between 1.2 for the electronic reader. These findings prove that low-cost electronics can match the results of commercial instrumentation using machine learning techniques.

## 1. Introduction

Pure glycerol, a colorless, odorless, viscous liquid with unique physical and chemical characteristics, is one of the most versatile organic molecules [1]. Glycerol can work as a humectant, sweetener, or even a solvent, and consequently, it is widely used in the pharmaceutical, food, and cosmetic industries. Moreover, glycerol is commonly used as a constituent or reactive element in synthesis reactions for the chemical industry [2,3]. In addition, glycerol is a crucial part of the structure of organic matter as the molecular base of fats or triglycerides, the main energy reservoir for animals and vegetables. However, pure glycerol is hard to extract and is usually diluted in water in a solution called glycerin, which is also soluble in alcohols. In contrast, it is insoluble in hydrocarbons and only partially soluble in many organic solvents.

On the other hand, crude glycerin (80%) is a primary by-product of biodiesel production from refined vegetable oils. Indeed, nowadays, this process is the principal source of pure glycerin (95–99%). However, the glycerin extracted must be separated and purified before being used for industrial applications [4]. Similarly, low-quality biodiesel due to inadequate purification involves some risk of critical engine issues, such as filter clogging or injector coking [5]. In conclusion, products with glycerin and biodiesel demand accurate and real-time sensing tools to characterize glycerin purity during refining. This paper aims to present a system for measuring glycerin solution permittivity with a Dielectric Resonator (DR) sensor excited by microwave signals created with off-the-shelf electronic components.

Microwave (MW) sensors are a cost-effective and adaptable solution for identifying and measuring substances. From an electronic point of view, MW sensors can be modeled as an RLC high-frequency circuit that functions as a notch filter with a high Q factor. These sensors are sensitive to changes in capacitance, leading to a shift in the resonance frequency [6]. The effective permittivity of the medium surrounding the DR is intimately related to the resonator capacitance. In sum, the DR is an indirect detector for substances with permittivity values greater than air, which is usually the reference medium [7]. This study aimed to detect changes in glycerin concentration by observing the effect on the DR resonance frequency, as each solution has a distinct complex permittivity value.

One of the main advantages of DR sensors is their straightforward fabrication, as they are usually made of a bulk dielectric material with a regular geometry, most commonly cylindrical. The material’s physical dimensions and relative permittivity tune its resonance behavior over a narrow frequency range [8]. In addition, a DR is an ideal MW radiating device with minimum conduction losses and high radiation efficiency on a 3D surface [9]. Moreover, DR sensors are an attractive choice for developing low-cost detection devices since they could be fully passive, avoiding the requirement of batteries to feed the sensor [10]. Other resonant technologies have been proposed for sensing liquids in the microwave region. In particular, split-ring resonators are the most popular sensors, as reported in several reviews in the last few years [7,11,12]. However, these technologies are primarily planar designs generally oriented for microfluidic or submersion applications since managing fluids demands a specific configuration. Therefore, a cylindrical DR with a small cavity for drop measurements could be a solid and practical alternative for liquid analysis.

Sensor calibration is crucial for obtaining accurate and valuable results. This process establishes a correlation between the DR sensor signal and the expected measurement value. Conventional calibration, which involves compiling a measurement dataset, selecting relevant variables, and performing statistical analysis [13], presents challenges and drawbacks that machine learning (ML) can address [14]. ML, a subfield of artificial intelligence, focuses on identifying complex patterns within data and then applying that knowledge to make predictions. The power of ML models to detect correlations inside these data has the potential to enhance sensor performance, especially in cases with a non-linear relationship between the analyzed process and the signal acquired [15]. For example, in this research study, the permittivity of binary mixtures follows a double Debye model since each solution component has a unique dielectric relaxation [16]. In addition, the calibration based on fixed parameters, such as resonance frequency, is less optimal and may introduce bias or variance, reducing the sensor’s performance since these parameters are not the complete signal information. In contrast, machine learning models pursue a balanced trade-off between variance and bias by considering the complete signal information. Our objective is to improve the sensitivity of DR sensors by applying ML techniques. We also aim to create a real-time sensing system capable of automatically detecting slight variations in permittivity, enabling us to determine the composition of the glycerin solution.

However, all the advantages of DR sensors are strongly reduced due to the electronic instrumentation systems for high-frequency measurements. For example, a Vector Network Analyzer (VNA) requires a high economic investment but also demands qualified personnel and complex calibration, and its bulky size hinders its use for automatic real-time measurements in industrial environments. In addition, the cost and complexity of high-end equipment diminish the market penetration of on-site analytical devices in other fields, such as environmental research [17] or diagnostic devices for point-of-care strategies [18], where DR sensors could be the base of numerous applications. It must be noted that these limitations have a more severe impact on low-income countries, where the need for low-cost sensors is more acute [19]. Therefore, it is necessary to reduce the cost and increase the portability of the electronic equipment to interface with DR sensors. In recent years, the development of the Internet of Things and the enthusiasm for open-source hardware and software have opened many possibilities for developing low-cost electronics using microcontrollers or microcomputers such as Arduino or Raspberry Pi [20]. These boards allow I/O control of signals within an embedded system. While commercial devices give strict specifications, open-hardware designs have the inherent advantage of customization, allowing the device to adapt to the needs of the analytical objective. We propose using an Arduino microcontroller and economic MW components to replace the VNA measurements at a more reasonable price but with comparable performance.

## 2. Materials and Methods

**Figure 1:**
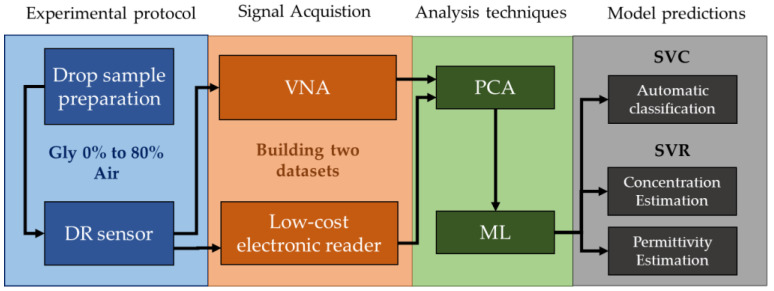
Summary of the experimental workflow.

This section provides a comprehensive description of each step of the DR sensor workflow (Figure 1). First, the DR overview and the experimental setup are presented, with a specific subsection explaining our novel electronic reader components and their functioning. Afterward, the data collection is described, including the measurement protocol and sample selection process. Finally, this section briefly introduces the ML learning techniques employed, with their respective parameters.

### 2.1. DR Sensor Overview

**Figure 2:**
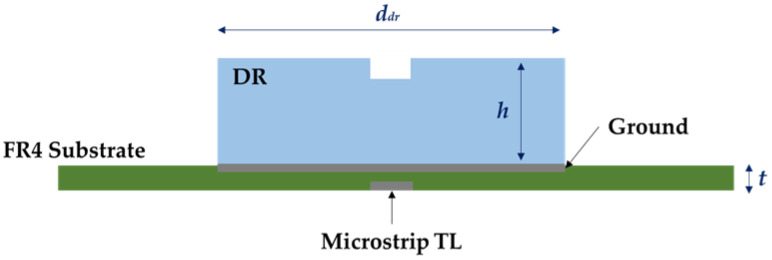
DR sensor scheme showing the sensor placement on the ground to achieve the coupling between the DR and the Microstrip TL.

The authors presented the DR sensor used in these experiments in a previous study involving the liquid characterization of water-ethanol binary mixtures [6]. As depicted in Figure 2, the DR design has a cylindrical structure with a radius d_dr_ = 34 mm and height h = 8 mm, fabricated using zirconia, a ceramic material with high relative permittivity, ε_r_ = 29, and minimal conduction losses. A small cylindrical analysis chamber is placed on the DR top for drop measurements. The sensor is fed through a microstrip transmission line (TL) adjusted to 50 Ω and connected to an SMA port. An optimized rectangular slot was etched on the ground plane to maximize energy transmission and achieve excellent coupling efficiency. 

**Figure 3:**
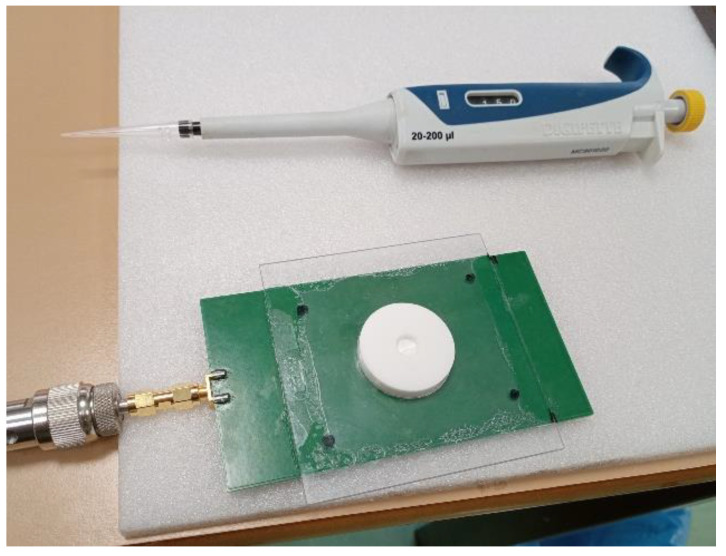
Experimental setup for the DR sensor with a polycarbonate supporting frame and the SMA connection to the VNA port.

Additionally, a polycarbonate frame was used to fix the DR to the FR4 fiberglass substrate (*ε_r_* = 4.4, t = 1.55 mm) to enable coupling stability and measurement reproducibility (Figure 3). The fundamental HEM_110_ mode is excited inside the DR with a theoretical resonance frequency related to the physical dimensions and material permittivity (1) [6]. Simulation results and experimental observations determined that the DR resonance frequency on air is 2.473 GHz. The frequency shift is due to the interaction between the electromagnetic fields inside the DR and the liquid sample inside the analysis chamber, as defined by the perturbation theory [21].

(1) $${\left(f_{r}\right)}_{110}=\frac{c}{{2r}_{dr}\pi \sqrt{\epsilon_{r}}}\left(1.71+\frac{r_{dr}}{h}+1.578{\left(\frac{r_{dr}}{2h}\right)}^{2}\right)$$

### 2.2. Electronic Instrumentation: From Vector Network Analyzer to Low-Cost Detection Devices

**Figure 4:**
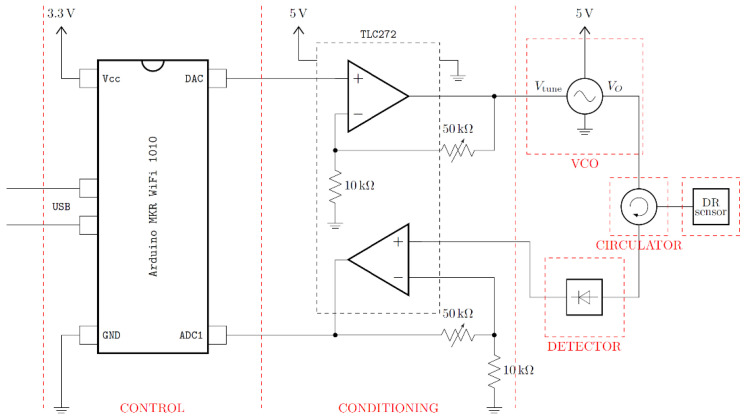
Electric circuit diagram of the electronic reader with all the components involved in the signal acquisition. This design is divided into three parts: control, conditioning, and MW sensing.

The main objective of this research study was to compare the DR sensor performance of an electronic instrumentation device and a novel electronic reader made with low-cost components. First, the spectra were obtained using the Anritsu MS46122B VNA calibrated in the frequency range of 2.25–2.55 GHz and with a sampling buffer of 5000 points. The 1-port mode configuration was employed to obtain the reflection coefficient |s11| and frequency values for the ML analysis. The VNA measures established a results baseline to compare with our designed portable electronic reader and validate its performance. The electronic reader is an improved design from previous research [22]. Figure 4 shows the electric circuit with the control, conditioning, and MW sensing modules. An Arduino MKR WiFi 1010 is the base for the control module for signal generation and acquisition.

**Figure 5:**
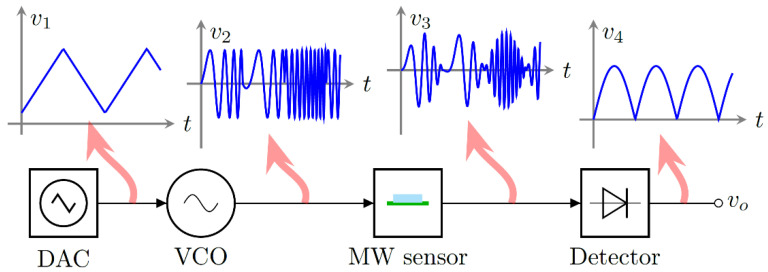
Signal transformation along the electronic reader workflow: v_1_ triangular signal from the DAC, v_2_ frequency sweep from the VCO, v_3_ reflected signal by the MW sensor, v_4_ acquired signal by the Arduino ADC.

The Arduino DAC generates a sweeping triangular signal (v_1_ in Figure 5), which is transmitted to the VCO (Minicircuits ZX95-2536C-S+) through a reconditioning circuit to adapt the input voltage for tuning the output MW signal within the 2.25–2.55 GHz range (v_2_ in Figure 5). The VCO is connected to a circulator (UiY CC2528A2400T2500SF) to manage the signal direction. Afterward, the MW signal is transmitted to the DR sensor by the SMA port. Similar to the VNA acquisition, the DR sensor works in reflection: the circulator receives back the reflected MW signal (v_3_ in Figure 5) and then transmits the signal to the amplification circuit to increase voltage resolution. Finally, the measured signal is transmitted to the power detector and then to the Arduino, which digitalizes the signal converting the power values into voltage units by timestamp (v_4_ in Figure 5). It must be noted that the VNA acquires a unique frequency sweep, while the electronic reader records a signal with several sweeps in the time domain. In addition, each electronic reader signal for the same glycerine concentration will be desynchronized, adding variance to the dataset.

### 2.3. Measurement Protocol

The experiments were performed with air and glycerin solutions that varied from 0% to 80% in 10% intervals (Table 1). Higher concentrations, such as 90% or pure glycerin (99%), were not included in the dataset as they were too viscous to ensure the accurate volume with a micropipette. The measurements were obtained by filling the resonator cavity with a 150 µL volume sample. The resulting VNA dataset includes 100 spectra acquired for each solution, while the electronic reader dataset contains 180 signals for every solution obtained from 35 drop samples with five-six repetitions each, with six seconds of delay in between. All signals acquired are available in a GitHub public repository (https://github.com/MigMH/VNA_ER_GlycerinSolutions accessed on 3 April 2023).

The relative permittivity values for the selected glycerin concentration were extracted from [23] in the 2.25–2.55 GHz frequency range at 21 °C, varying from 17 to 74.32. In contrast, the permittivity value for pure water is 78.3 at 20 °C [24]. In order to validate these experimental results, the permittivity of each solution was estimated using the Maxwell–Garnett Mixing Rule (2), where *ε_sol_* and *ε_wat_* are the dielectric constants of solvent and water, respectively, and |m| is the volumetric fraction of the solvent solution in water [25]. Both permittivity values are drastically different (Table 1); a priori, it is not feasible to determine if the formula fits these experimental data or if this data is accurate. Unfortunately, to the author’s knowledge, there are no more references for this glycerin concentration in the frequency working range. Moreover, other authors [26] measured the same concentrations with a differential microwave sensor using the same reference permittivity values but at 1.56 GHz. Therefore, as a side objective, this article aims to use the VNA and our low-cost electronic reader to test which permittivity values fit better with the ML models.

(2) $$\epsilon_{eff}=\epsilon_{sol}+3\left|m\right|\epsilon_{sol}\frac{\epsilon_{wat}-\epsilon_{sol}}{\epsilon_{wat}+2\epsilon_{sol}-\left|m\right|\left(\epsilon_{wat}-\epsilon_{sol}\right)}\to \left|m\right|=\frac{V_{sol}}{V_{wat}}$$

### 2.4. Analysis Techniques

Implementing ML models demands feature reduction methods due to the high dimensionality of the obtained spectral data. For this reason, Principal Component Analysis (PCA) is employed, a statistical algorithm that identifies the directions of maximum variability within the data structure and generates a new mathematical space where each spectrum is projected while retaining essential information [27]. Each spectrum is defined by a vector in the principal components obtained from PCA [28], and this vector serves as the input feature for an ML model. The next step was labeling these data with their corresponding class to perform supervised learning; several models were tested in a preliminary study, such as Random Forest or XGBoost, but Support Vector Machine (SVM) achieved slightly better performance. This algorithm traces a hyperplane in the data space to classify each sample, and it is calculated to maximize the separation between classes [29]. The support vectors are the closest data points of each class to the decision boundary, and since SVM only requires a small number of support vectors to define the hyperplane, it is a reliable method for working with small datasets [30]. This is a considerable advantage since acquiring a huge dataset is significantly time-consuming. In addition, SVM is a versatile model, and it can be used for regression as well as classification; in this case, the model prediction is an estimate of permittivity. The SVM hyperparameters were chosen using Bayesian optimization: C, which defines the hyperplane exclusion margin, and γ, which regulates the influence distance of a single training point (Table 2). Finally, in order to prevent overfitting, the dataset was divided into a training set (70%) and a test set (30%). The K-fold Cross-Validation method was applied during the model training with k = 5 folds [31].

## 3. Results and Discussion

The main purpose of this section is to compare the ML performance comparison between the datasets acquired by the VNA and by our low-cost electronic reader. Firstly, we analyze the signal characterization of each device, with different glycerin solutions. Secondly, we show how the information within these signals is condensed and projected using PCA to improve the interpretation. Thirdly, we evaluate and compare the performance of ML models for the automatic classification of each glycerin concentration. Finally, we estimate the solution permittivity and validate the results with two different sources: the experimental data from [23] and the Maxwell–Garnett mixing rule.

### 3.1. Signal Characterization

**Figure 6:**
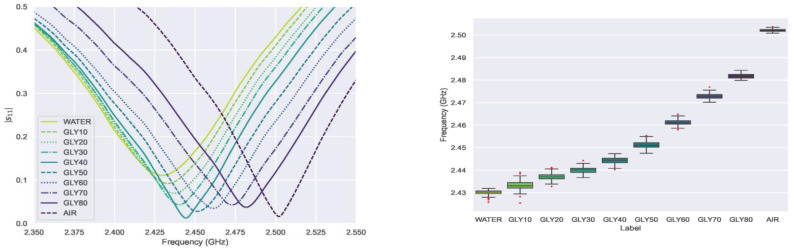
VNA average signals (**left**) and boxplot of the resonance peak distribution (**right**) for each glycerin concentration.

The average spectra of each solution in Figure 6 indicate drastic resonance frequency changes. Lower glycerin concentrations result in a reduction in the resonance frequency since the higher volume of water increases the solution permittivity. As glycerin concentration increases, the maximum |s_11_| amplitude rises until Gly40%, which approaches the value of air. Beyond this concentration, the |s_11_| amplitude gradually decreases. This change could be related to the complex interaction between the dielectric relaxation of glycerol and water. Given that the loss factor affects the resonance amplitude, the DR sensor might actually be measuring complex permittivity. Further research using alternative dielectric characterization methods would help to acquire a reliable reference to compare with our sensor results.

**Figure 7:**
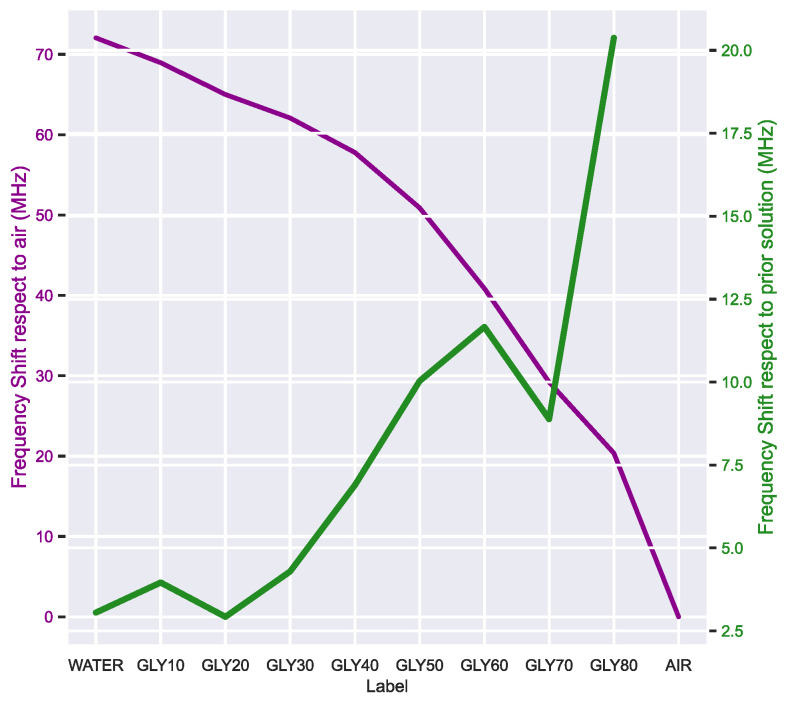
Frequency shift variation with respect to the air reference value (purple) and with respect to the previous glycerin concentration (green). The trend is non-linear since the frequency shift is more significant for values close to air and more uniform for values close to water.

The resonance peak distribution was analyzed with a boxplot, where the average value and the dispersion for each concentration can be easily compared. Figure 6 shows a minor overlap between the low glycerin concentrations, which diminishes as the water volume fraction decreases. Indeed, with concentrations of 40% glycerin and above, each class peak distribution is entirely isolated. Compared to the previous study of this DR design with water-ethanol solutions [6], the variance in the concentration has been drastically reduced thanks to improvements in the setup and the measurement protocol. According to these findings, neither the solution permittivity nor the frequency shift (Figure 7) evolves linearly, in accordance with both the Maxwell–Garnett Mixing Rule and the data extracted from [23] (Table 1). The resonance behavior of the sensor itself is another factor that helps to explain this resonance peak distribution; since the resonance frequency shift is a non-linear function of permittivity. As shown in Figure 7, the frequency shift is more pronounced when the solution permittivity is closer to the reference (ε_r_ = 1); in other words, the shift increment reduces as the glycerin concentration rises. Both factors seem to impact the frequency shift significantly and must be considered for future research, especially with low glycerin concentrations. These effects are far less significant in high concentrations, such as crude glycerin from biodiesel refining. Other authors in [26] propose a differential microwave sensor for the same glycerin concentrations, but their results are hardly comparable since the sensor design is entirely different and relies on a microfluidic channel with a pumping system. Although they achieve good sensitivity, their measurements are based solely on one parameter, the amplitude change in differential signal |s^DC^_11_|. The purpose of this paper is not to limit the sensor sensitivity analysis to a single parameter, the resonance peak shift, but to apply ML to study how the whole signal is affected. Given that the sensitivity is not a constant since it depends on the resonators’ non-linear behavior, the accuracy of the ML models would represent a more useful indicator of the DR sensing capability.

**Figure 8:**
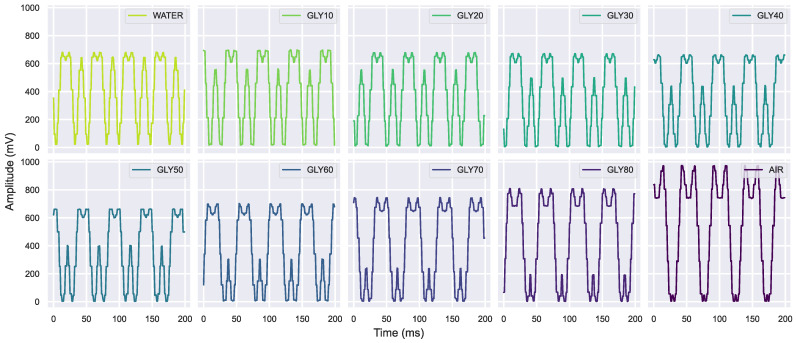
The electronic reader signals examples for each glycerin concentration and air.

The periodic signal by the electronic reader resembles a rectified sum of two sinusoidal waves. It shows clear differences when the glycerin concentration changes (Figure 8), but the interpretation is less intuitive than the case of VNA spectra, where the concentration can be guessed by looking at the peak frequency shift. Nevertheless, it is possible to spot a pattern; one of the waves has practically vanished in the air signal but is increasing with the glycerin concentration, reaching the maximum for water. Therefore, this wave corresponds to lower frequencies closer to the 2.25 GHz margin detected by the reader (Figure 6). The second wave diminishes from its maximum in the air until remaining steady around Gly 40%; thus, this wave corresponds to higher frequencies around the 2.55 GHz margin. However, all the voltage amplitude changes are particularly subtle between glycerin solutions with similar concentrations. Consequently, the electronic reader signals would be less helpful in analyzing glycerin solutions without applying PCA or ML.

### 3.2. Principal Component Analysis (PCA)

**Figure 9:**
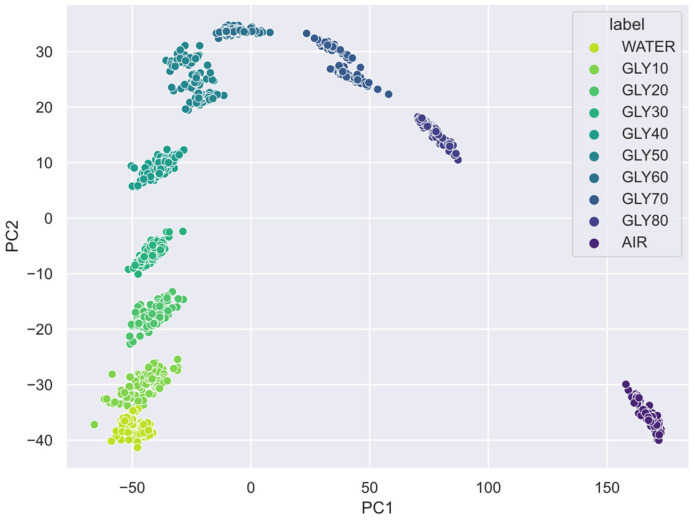
PCA scatter plot of the VNA spectra.

Only two principal components were required for the PCA to condense 99% of the information within the VNA spectra data. Since the PCA projects the signals into an abstract space, its explainability is typically low. However, in this case, the PCA plot for the VNA reveals discrete and separate clusters in what seems to be a concentration curve (Figure 9). When using PCA, the whole signal is considered rather than just the resonance peak. Therefore, it is simple to distinguish between each glycerin solution without the minor overlap mentioned before in the boxplot analysis (Figure 6). In conclusion, the PCA increases the class separability of these spectral data by reducing data dimensions.

**Figure 10:**
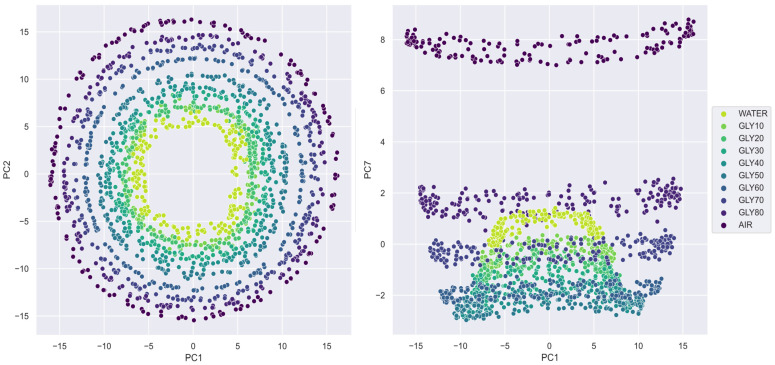
PCA scatter plots of ER signals.

Because the electronic reader generates desynchronized periodic signals, the PCA demands seven principal components to reach 97% of the explained variance, as opposed to the PC required by the VNA. VNA projections show clear and distinct clusters (Figure 9), while the electronic reader projects circular patterns (Figure 10). In particular, the PC1-PC2 plot shows a projection grouped in concentric circles, each corresponding to a glycerin concentration, in what appears to be a graduation of permittivity. It must be noted that the class separability reaches its maximum when considering all the dimensions at once. For example, the PC1-PC7 plot extends the graduation in another dimension where the solution differences are incremented. In summary, both acquisition methods show good class separability in the PCA plots that anticipate positive results in the ML models. For this reason, the lack of synchronization is very useful to add variability in the model learning since it reduces bias, but at the cost of increasing the complexity of the SVM model.

### 3.3. Glycerin Classification

**Figure 11:**
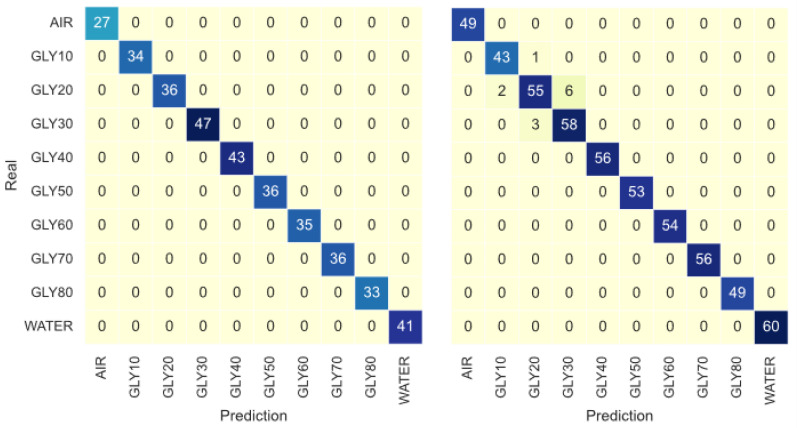
Confusion Matrix for the SVM classification model for the VNA signals (**left**) and Electronic Reader signal (**right**).

The SVM classification model achieved excellent accuracy (around 97–99%) for both the VNA and the electronic reader (Table 3), in accordance with the insights from the PCA. The confusion matrixes in Figure 11 show barely any contamination between classes. In contrast, the regression models show some performance differences between both acquisition methods; the Root Mean Squared Error (RMSE) for the electronic reader is between two and three times bigger than the VNA (Table 3). To the author’s knowledge, there are no similar experiments measuring glycerin concentration with dielectric resonators or automatic classification of substances with microwave sensors. However, recent ML applications for electrochemical and optical biosensors using PCA and SVM have shown results in accordance with this research [14]. Therefore, these findings reinforce the value of DR sensors and ML models for classifying glycerin solutions.

### 3.4. Permittivity Estimation

**Figure 12:**
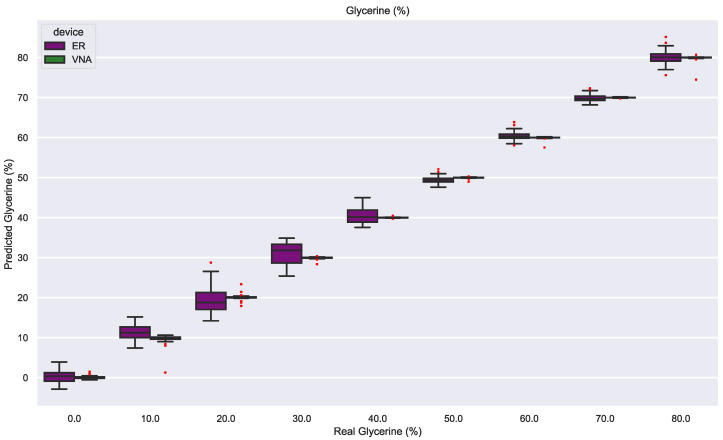
Regression model predictions for glycerin (%).

The glycerin concentrations are labeled as discrete values for the SVM classification model. However, the Support Vector Regressor (SVR) can be employed to estimate the glycerin as a continuous variable after relabeling the training dataset and removing the air samples. The model performance is almost perfect for the VNA with an RMSE of 0.70% and highly favorable for the electronic reader with an RMSE of 1.93% (Table 3). The boxplot comparison between both devices in Figure 12 shows that the VNA permittivity estimations fit the real concentration almost perfectly: the box is almost a flat line, and the primary source of RMSE is some outliers. In contrast, the electronic reader estimations are more scattered around the original value since the variance is greater, especially with glycerin concentrations from 10% to 40%. This result concurs with the PCA plots from the electronic reader (Figure 9 and Figure 10), where the distance between these concentrations is minimal. As previously stated, the DR sensor’s non-linear response could reduce sensitivity when the permittivity sample is significantly higher than air permittivity, as occurs with low glycerin concentrations. However, for concentrations greater than 40%, the electronic reader performance is more precise and similar to the VNA performance.

**Figure 13:**
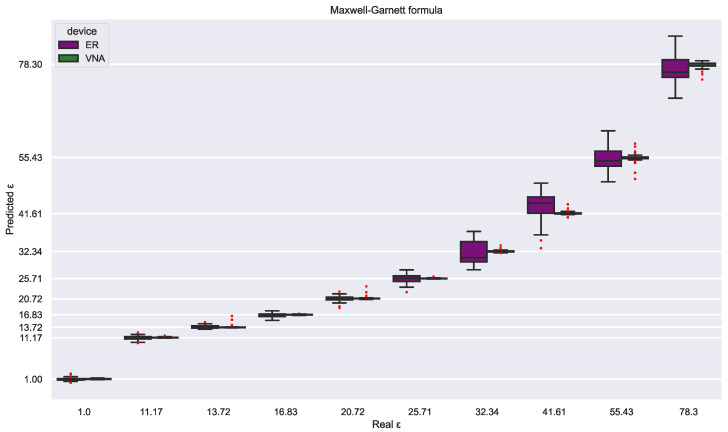
Permittivity estimations for both devices from an SVR model trained with the permittivity values from the Maxwell–Garnett mixing rule.

**Figure 14:**
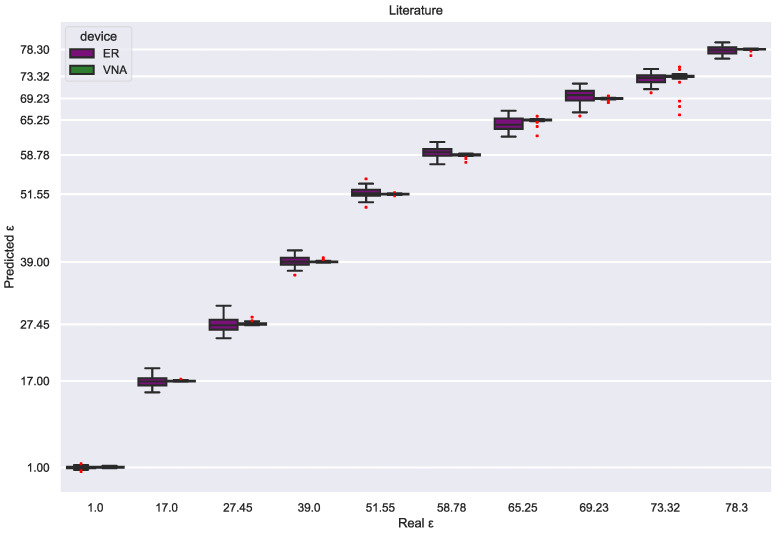
Permittivity estimations for both devices from an SVR model trained with the permittivity values found in the literature [23].

Additionally, two further SVR models were trained to estimate solution permittivity rather than glycerin concentration. Each model was trained with two permittivity sources: the Maxwell–Garnett Mixing Rule [25] and the values acquired by dielectric spectroscopy from the literature [23] (Table 1). The VNA achieved excellent and identical performance with an RMSE close to 0.60 (Table 3) for both permittivity sources (Figure 13 and Figure 14). However, the VNA dataset hinders the evaluation of the reliability of both permittivity sources since the excellent class separability helps the SVR models estimate the solution permittivity independently of the value distribution.

On the other hand, the electronic reader is less precise and more sensitive to perturbations; therefore, both SVR models show distinct estimation patterns. First, the estimations based on the Maxwell–Garnett mixing rule are almost perfect for low permittivity values, while for high permittivity, the error is drastically increased (Figure 13). This contrasts clearly with the SVR model trained with the permittivity values from [23] since these estimations have a more uniform error distribution by class (Figure 14), which are comparable to the VNAs in all the permittivity ranges. This difference is reflected in the RMSE, 1.119 for the literature SVR model and 2.091 for the Maxwell–Garnett mixing rule SVR model. In sum, the experimental values seem correct but may be affected by minor experimental errors. In contrast, the Maxwell–Garnett mixing rule, which is commonly used to estimate the permittivity of composite materials with inclusions [32], is probably not optimal for liquid mixtures. This result indicates that the combination of the electronic reader and SVR models is a good method to evaluate the reliability of mixing formulas or experimental dielectric characterization.

## 4. Conclusions

This study proposed using a dielectric resonator (DR) sensor to determine glycerin solution concentration. A commercial VNA was used to establish a benchmark for the sensor performance and, thus, validate our designed electronic device. Measurements of air and nine different glycerin concentrations were taken within a relative permittivity range of 1 to 78.3. Using Support Vector Machine (SVM) and Principal Component Analysis (PCA), both devices obtained outstanding accuracy (98–100%). Additionally, permittivity estimation using the Support Vector Regressor (SVR) accomplished low RMSE values between 0.6 and 1.2. These results prove that by applying ML with the proposed workflow (Figure 1), low-cost portable electronics can achieve comparable results to complex electronic instrumentation equipment. This significant contribution opens many opportunities for future developments since this workflow is not limited to our DR design and could be applied to any sensor. These findings also emphasize the DR sensor’s capacity to generate accurate permittivity estimations. Consequently, if the SVM model is trained with the correct permittivity data, it could estimate the permittivity of any liquid inside its cavity chamber. Moreover, this methodology is able to validate any permittivity characterization, including mixing formulas, which we consider critical since there is a lack of dielectric characterization data for many sensing applications, especially if the sensor work in a narrow frequency range. In conclusion, the designed DR sensor and the workflow proposed is a promising combination for crude glycerin analysis in all sectors where glycerin is required. ML models offer a precise method for analyzing the sensor inputs, making them the ideal support technology for the DR sensor. In future works, this methodology will be adapted to new analytical targets and expanded to reach the full potential of this technology.

## Figures and Tables

**Table 1:** List of glycerin solutions tested with their relative permittivity values at 20–21 °C.

	*ε_r_* Maxwell–Garnett Mixing Rule	*ε_r_* Literature
Air	1	1
Gly80%	11.17	17.00
Gly70%	13.72	27.45
Gly60%	16.83	39.00
Gly50%	20.72	51.55
Gly40%	25.71	58.78
Gly30%	32.34	65.25
Gly20%	41.61	69.23
Gly10%	55.43	74.32
Water	78.30	78.30

**Table 2:** ML models hyperparameters for the VNA and the electronic reader (ER).

Model	C	γ
VNA Classification	2620	0.00087
VNA Regression glycerin (%)	4000	0.01224
VNA Regression mixing rule values	4000	0.01362
VNA Regression literature values	4540	0.01243
ER Classification	4197	0.00100
ER Regression glycerin (%)	10,000	0.00323
ER Regression mixing rule values	9357	0.00623
ER Regression literature values	8247	0.00288

**Table 3:** ML performance results.

Models	VNA Accuracy	ER Accuracy
SVM	99.33%	97.41%
	**VNA RMSE**	**ER RMSE**
SVR Glycerin (%)	0.70%	1.93%
SVR mixing rule values	0.629	2.091
SVR literature values	0.599	1.119

## Data Availability

All signals acquired are available in a GitHub public repository (https://github.com/MigMH/VNA_ER_GlycerinSolutions accessed on 3 April 2023).
